# Protocol optimization of a targeted sequencing panel for genomic profiling of bronchoalveolar lavage fluid in lung cancer

**DOI:** 10.1002/cam4.6380

**Published:** 2023-08-17

**Authors:** Cassandra L. Sather, Pamela Yang, Chaomei Zhang, Matthew P. Fitzgibbon, Michelle Fournier, Eric Toloza, Amit Tandon, Matthew Schabath, Sean Yoder, Viswam S. Nair

**Affiliations:** ^1^ Shared Resources Fred Hutchinson Cancer Center Seattle Washington USA; ^2^ Tissue and Molecular Genomics Cores H. Lee Moffitt Cancer Center & Research Institute Tampa Florida USA; ^3^ Department of Thoracic Oncology H. Lee Moffitt Cancer Center & Research Institute Tampa Florida USA; ^4^ Department of Cancer Epidemiology H. Lee Moffitt Cancer Center & Research Institute Tampa Florida USA; ^5^ Division of Pulmonary, Critical Care & Sleep Medicine University of Washington Seattle Washington USA; ^6^ Clinical Research Division Fred Hutchinson Cancer Center Seattle Washington USA; ^7^ Present address: Advent Health Wesley Chapel Florida USA

**Keywords:** AVENIO®, BAL, CAPP‐Seq, genomics, lung cancer

## Abstract

**Introduction:**

We investigated a commercially available sequencing panel to study the effect of sequencing depth, variant calling strategy, and targeted sequencing region on identifying tumor‐derived variants in cell‐free bronchoalveolar lavage (cfBAL) DNA compared with plasma cfDNA.

**Methods:**

Sequencing was performed at low or high coverage using two filtering algorithms to identify tumor variants on two panels targeting 77 and 197 genes respectively.

**Results:**

One hundred and four sequencing files from 40 matched DNA samples of cfBAL, plasma, germline leukocytes, and archival tumor specimens in 10 patients with early‐stage lung cancer were analyzed. By low‐coverage sequencing, tumor‐derived cfBAL variants were detected in 5/10 patients (50%) compared with 2/10 (20%) for plasma. High‐coverage sequencing did not affect the number of tumor‐derived variants detected in either biospecimen type. Accounting for germline mutations eliminated false‐positive plasma calls regardless of coverage (0/10 patients with tumor‐derived variants identified) and increased the number of cfBAL calls (5/10 patients with tumor‐derived variants identified). These results were not affected by the number of targeted genes.

## Graphical abstract

Bronchoalveolar lavage fluid identifies more tumor‐derived variants than plasma in early‐stage lung cancer using a commercially available, targeted hybrid‐capture panel. Calling strategy affects the identification of variants more than sequencing depth or targeted sequencing region using these panels. Clonal hematopoiesis results in false positive calls in plasma using these panels.

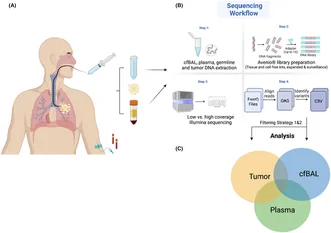

## 1 INTRODUCTION

Detecting lung cancer by blood liquid biopsy using genomic profiling has advanced dramatically in the past 10 years.^1^, ^2^ There are now several clinical options that are being utilized in medical practice to profile advanced‐stage disease in blood for targeted therapy, or to assess the possibility of clinical trial eligibility. However, the performance of genomic profiling drops off drastically for earlier stage disease.^3^, ^4^, ^5^

Currently, there are many trials investigating the utility of targeted therapy or immunotherapy in the neoadjuvant or adjuvant setting for early‐stage lung cancer. This has resulted in several paradigm‐altering therapies that include EGFR inhibition instead of traditional chemotherapy after surgically resected disease and, more recently, neoadjuvant immunotherapy prior to surgical resection in Stage Ib‐III disease.^6^, ^7^ Methods to improve our ability to track and profile genomic alterations in early‐stage lung cancer from biopsies are therefore mandated.

We previously showed that profiling cell‐free bronchoalveolar lavage (cfBAL) fluid obtained from bronchoscopy during lung cancer evaluation augments tumor mutation detection and can potentially assist with lung cancer diagnosis using CAPP‐Seq.^8^ Here, we follow up on these findings and study the effect of sequencing depth, variant filtering strategy, and genomic target regions on cfBAL profiling using a commercial assay based on the CAPP‐Seq platform. A secondary goal of the study was to demonstrate that utilizing a commercial product to identify mutations calls without the need for bioinformatics expertise can democratize BAL profiling for clinical implementation.

## 2 MATERIALS AND METHODS

### 2.1 Enrollment

**FIGURE 1:**
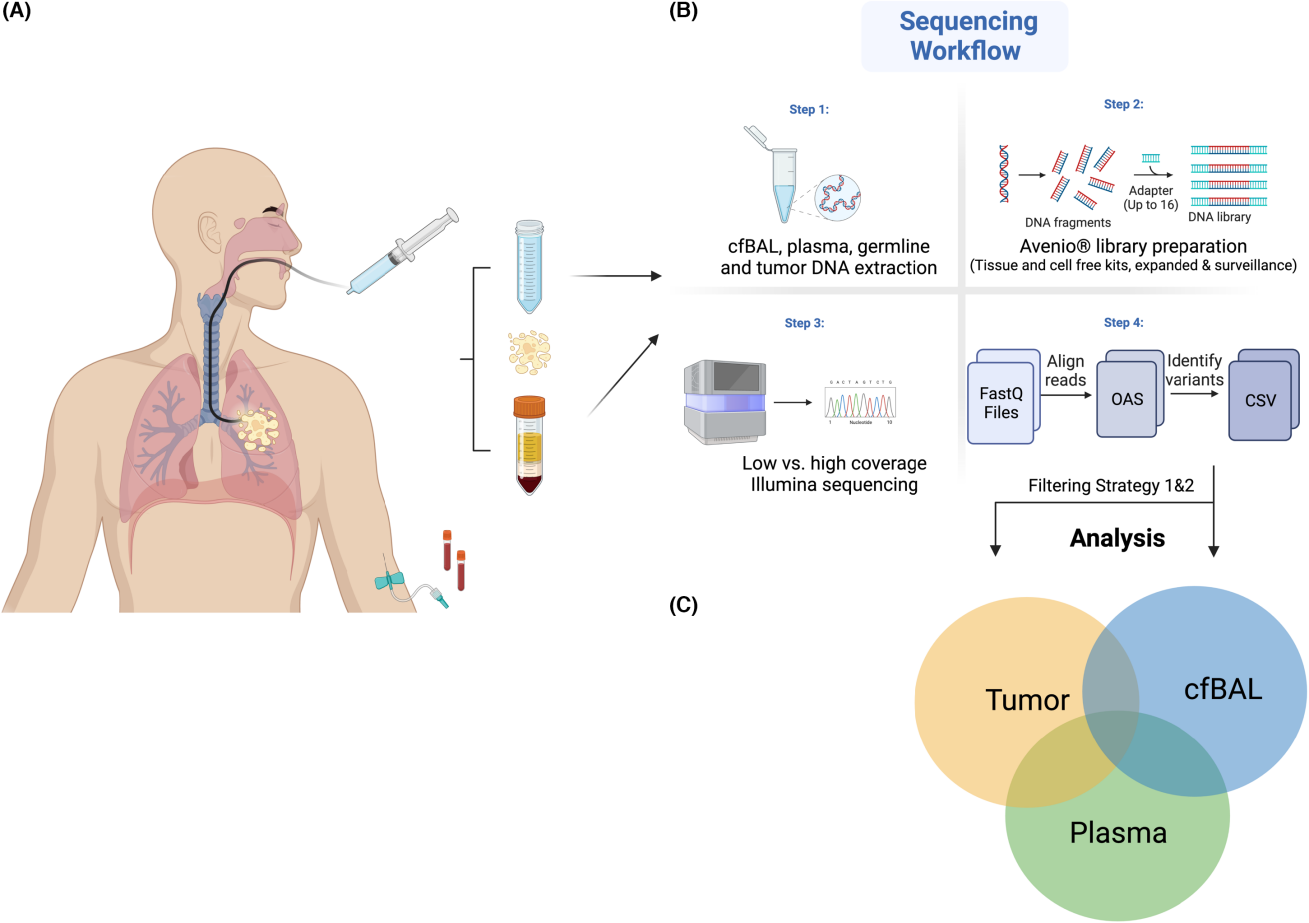
Study overview. (A) A bronchoalveolar lavage of normal saline was directed toward the lung tumor. The supernatant of the retrieved fluid was separated and stored for extraction to cell‐free BAL (cfBAL) DNA. Blood drawn prior to BAL was separated into plasma‐depleted leukocytes and cell‐free plasma and stored for extraction to germline DNA and plasma cfDNA, respectively. Archival tumor blocks from resected cancers were sectioned for extraction to tumor DNA. (B) Cell‐free and tumor kits were utilized for library preparation prior to sequencing at a goal of 50 (low‐coverage sequencing) to 100 million (high‐coverage sequencing) total reads using Illumina sequencers. FastQ files were uploaded to a local server and analyzed using the installed Oncology Analysis Software (OAS, v 2.0) to identify variants for plasma cfDNA and cfBAL DNA. The OAS calling strategy (Filtering Strategy 1, FS 1) versus one based on subtracting germline variants (Filtering Strategy 2, FS 2) was employed to identify variants. (C) Tumor variants were compared with plasma and cfBAL variants using low vs. high‐coverage sequencing and FS 1 versus FS 2. Image created at BioRender.com.

This was a single‐center, observational study of genomic profiling in bronchoalveolar lavage (BAL) fluid from patients enrolled in 2018–2019 (Data S1). A targeted BAL in the segment or subsegment of the tumor was obtained during a clinically indicated bronchoscopy or surgical resection for lung cancer diagnosis or treatment (Figure 1). Blood was obtained prior to intervention for germline DNA and plasma sequencing. Archival specimens from formalin‐fixed paraffin embedded primary tumor blocks were utilized for comparison to bio‐fluids. All research was IRB approved, and informed written patient consent was obtained before enrollment and prior to sample collection.

### 2.2 Sample processing

Cell‐free BAL, plasma, leukocytes, and tumor sections were extracted using standard kits (Data S1). Genomic and tumor DNA were quantified and checked for quality by spectrofluorometry using a Qubit fluorometer (Thermo Fisher Scientific). Cell‐free BAL DNA and plasma cfDNA were quantified and checked for quality using the Cell‐Free DNA ScreenTape Analysis on the 4200 TapeStation instrument (Agilent Technologies). Fragmentation by sonication (Covaris) was performed on cfBAL samples with or without DNA clean‐up. Plasma cfDNA was carried forward without further processing unless genomic clean‐up was required based on inspection of the DNA traces by bioanalyzer. Samples with ≥10 ng of cfDNA based on inspection of the DNA traces were carried forward for library preparation. Libraries for plasma cfDNA, cfBAL DNA, and germline DNA from leukocytes were prepared using the selected cell‐free AVENIO® kit (Roche, Basel, Switzerland) according to the manufacturer's protocol. Tumor libraries were prepared after confirming the input amount required based on the Q‐Ratio derived from the selected AVENIO® Tumor DNA Isolation and QC kit.

### 2.3 Sequencing

We hypothesized that use of the expanded panel would allow us to detect the largest number of cancer‐associated variants with the lowest amount of input materials and practical sequencing depths based on the manufacturer's technical performance reports (Data S1). The cell‐free and tumor‐expanded panel kits share identical target regions covering 77 genes spanning 192 kb based on hybrid‐capture technology (Table S1). After confirming adequate library preparation by TapeStation (Agilent), samples were carried forward to sequencing using HiSeq 2500, NextSeq 550, or HiSeqX (Illumina) sequencers in 150‐bp paired‐end read mode. We targeted 50 million total reads (referred to as *low‐coverage* sequencing from here) and 100 million total reads (referred to as *high‐coverage* sequencing from here) per sample based on the manufacturer's recommendations and existing literature.^7^, ^9^, ^10^ Fastq files were transferred from the sequencer to an on‐site server hosting the AVENIO® Oncology Analysis Software (Roche, OAS v2.0), which performed alignment and variant calling, with variants summarized as downloadable .csv files for analysis. (Figure 1, Data S1). Follow‐up sequencing using the surveillance panel targeting 197 genes spanning 198 kb was performed using the appropriate kits with the same library preparation and high‐coverage sequencing as described above (Table S1).

### 2.4 Tumor variant analysis

We utilized two strategies to analyze the data generated from the OAS (Figure 1). We first used the default filters applied by the Roche OAS to generate filtered variants, termed Filtering Strategy 1 (FS 1) from here.^9^ We then utilized germline DNA files to match variants with tumor, plasma, and BAL unfiltered variant lists. We removed any variants that overlapped and then proceeded to analyze variants identified in tumor and cell‐free compartments, termed Filtering Strategy 2 (FS 2) from here. Variants were analyzed qualitatively (number identified), and quantitatively (% Variant Allele Fraction [%VAF]) across biospecimens to assess whether cfBAL DNA identified more tumor‐derived variants than plasma cfDNA.

### 2.5 Statistical analysis

Statistical comparisons between fluid types or patient groups were made based on the underlying distribution of paired or unpaired datasets. Specific tests utilized are indicated in the results below when a statistic is reported.

## 3 RESULTS

In total, we analyzed 104 sequencing files from 40 matched cfBAL, plasma, germline, and tumor biospecimens in 10 patients with early‐stage lung cancer. The average age of these patients was 70 ± 8 years, 9 of 10 were past or current smokers (90%), and 6 out of 10 (60%) were male. Since BAL may be more sensitive for identifying tumor variants compared with blood,^8^ we analyzed early‐stage cancers (stage I = 6, stage II = 3, stage III = 1) for this study, of which nine (90%) were adenocarcinoma (Table S2).

All tumors from extracted paraffin were adequate for downstream library generation with input masses ranging from 30 to 54 ng based on Q‐Ratios (Table S3). A median of 4.8 mL of BAL fluid and 4.1 mL of plasma was utilized from patient samples studied (*p* = 0.11, Wilcoxon signed rank test [WSRT], Figure S1A) resulting in a median of 33.1 ng of cfBAL DNA and 50.0 ng of plasma cfDNA (*p* = 0.21, WSRT, Figure S1B). The median fragment size sequenced for cfBAL DNA (183 bp) was larger than plasma cfDNA (176 bp, *P*‐value = 0.15, WSRT, Figure S1C). Despite a lower input volume and mass input for library preparation, low‐coverage sequencing of cfBAL DNA resulted in more total and unique reads than plasma cfDNA (median unique depth 3319 vs. 3062, *p*‐value = 0.24, WSRT, Figure S1D,E).

**FIGURE 2:**
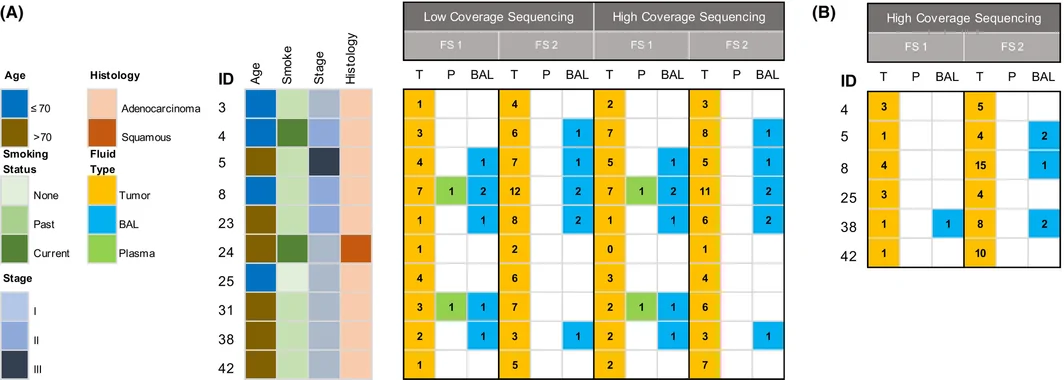
Variants identified by biospecimen type, sequencing coverage, filtering strategy, and panel. (A) Samples profiled on the expanded panel. Variant map shows tumor variants (gold) with matching plasma cfDNA (green) and cfBAL DNA (blue) variants. The number of variants associated with each sample type by patient is indicated within each box. Data are displayed by sequencing coverage and filtering strategy on the X‐axis (FS 1 = Filtering Strategy 1. FS 2 = Filtering Strategy 2). Patient characteristics (age, smoking history, stage, and histology) are displayed on the Y‐axis. (B) Samples profiled on the surveillance panel at high coverage. (Table S11). T = Tumor. P = Plasma. [Correction added on September 8, 2023 after first online publication. The figure 2 has been updated in this version.]

At ~9000× coverage, a total of 27, 35, and 10 (median of 2.5, 2, and 1 per patient) variants were identified in tumor DNA, cfBAL DNA, and plasma cfDNA, respectively, by low‐coverage sequencing using FS 1 (Figure S1F; Table S4). Notably, 16 filtered variants were detected in germline DNA. Matched to tumor, six tumor‐derived variants were identified in cfBAL DNA (50% of patients), and two tumor‐derived variants were identified in plasma cfDNA (20% of patients) (Figure 2A, Table S5).

As expected, with high‐coverage sequencing (~20,000×), the average unique median depth for tumor, plasma, and cfBAL was significantly higher compared with low‐coverage sequencing across these 10 patients (*p* = 0.003, 0.002, 0.015, respectively, WSRT, Tables S6 and S7). No additional tumor‐derived variants were identified in cfBAL DNA (*n* = 6) or plasma cfDNA (*n* = 2) by high‐coverage sequencing using FS 1 (Figure 2A; Table S8).

FS 2 identified one additional tumor‐derived variant call in cfBAL DNA in five patients total (50% of the cohort) regardless of sequencing coverage (Figure 2A; Table S5,8). Cell‐free BAL DNA %VAF of these tumor‐derived variants ranged from 0.07 to 0.85% (Table S9). Additionally, FS 2 eliminated calls in two patients with plasma variants identified by FS 1 presumably due to clonal hematopoiesis (CH), and no additional plasma variants were identified with high‐coverage sequencing (Figure 2A; Table S5,8).

To further explore whether our results were dependent on target region, we re‐sequenced six patient samples using the surveillance panel for cell‐free and tumor biospecimens. Cell‐free total mapped reads were comparable to the expanded panel using the high coverage approach, but cfBAL DNA (*P*‐value = 0.03) and plasma cfDNA (*P*‐value = 0.046) had a significantly greater unique read depth with the surveillance panel (Figure S2; Table S10). FS 1 yielded zero tumor‐derived calls in plasma cfDNA (0%), while one patient had tumor‐derived variants identified in cfBAL DNA (17%). FS 2 also yielded zero tumor‐derived calls in plasma (0%), but it identified three patients with cfBAL DNA tumor‐derived variants (50%) (Figure 2B; Table S11).

## 4 DISCUSSION

Here, we have extended our initial work examining the feasibility of profiling cell‐free BAL (cfBAL) in lung cancer by using a commercial platform to analyze 104 sequencing files from 40 samples in 10 lung cancer patients. We confirm that hybrid‐capture deep sequencing of cfBAL DNA is a useful adjunct to profile lung cancer and can resolve tumor variants in cfBAL DNA on the order of 0.1% VAF. Additionally, we demonstrate that cfBAL DNA adds value over plasma cfDNA by identifying tumor‐derived variants in half of patients from an early‐stage non‐small cell lung cancer (NSCLC) cohort where plasma detection is technically challenging, and lower sequencing depths may be utilized here for more cost‐effective sequencing. This is biologically plausible since proximal fluids are enriched in tumor‐associated biomarkers due to anatomic location, and the cell‐free portion removes contaminating “normal” genomes so rare variants can be detected. Further study in early‐stage lung cancer patients is required to confirm this finding over time.

The results also indicate that a filtering strategy accounting for germline mutations affects biospecimen mutation calls by reducing false positives in plasma cfDNA and increasing true positive calls in cfBAL DNA. Accounting for germline mutations is increasingly being recognized as a more precise method to identify tumor variant calling at the patient level in lieu of using publicly available databases.^3^ This, along with the default filtering strategy from the OAS defined in the supplemental methods, potentially explains the differences in FS1 and FS2 results for plasma and BAL. Finally, cfBAL DNA identified more tumor‐derived variants than plasma independent of hybrid‐capture panel type, indicating that our results may be generalizable to other sequencing assays with different targeted regions.

Although this study is limited by sample size, we corroborate previous work on BAL genomic profiling^8^ that suggests additional value to blood liquid biopsy in the pre‐surgical setting where a endoscopic biopsy may inform treatment decisions.^6^ BAL genomic profiling may provide useful information in recurrent or progressive disease as well, but additional study is required in these types of patients. While the use of a commercial assay facilitates implementation in other laboratory settings, caveats to implementation include (a) the need to account for germline variants during call identification (b) understanding that targeted sequencing region of the AVENIO® assay is not strictly designed to study NSCLC and (c) end‐to‐end research costs of sequencing per sample that ranged from $700–1200 US dollars for this study.

## AUTHOR CONTRIBUTIONS

**Cassandra L. Sather:** Data curation (equal); formal analysis (equal); investigation (equal); methodology (equal); project administration (equal); resources (equal); supervision (equal); writing – original draft (equal); writing – review and editing (equal). **Pamela Yang:** Data curation (supporting); methodology (supporting); resources (supporting); writing – review and editing (supporting). **Chaomei Zhang:** Data curation (supporting); methodology (supporting); resources (supporting); writing – review and editing (supporting). **Matthew P. Fitzgibbon:** Data curation (supporting); formal analysis (supporting); resources (supporting); software (supporting); visualization (supporting); writing – review and editing (supporting). **Michelle Fournier:** Project administration (supporting); resources (supporting); writing – review and editing (supporting). **Eric Toloza:** Data curation (supporting); project administration (supporting); resources (supporting); supervision (supporting); writing – review and editing (supporting). **Amit Tandon:** Data curation (supporting); project administration (supporting); resources (supporting); supervision (supporting); writing – review and editing (supporting). **Matthew B. Schabath:** Data curation (supporting); project administration (supporting); resources (supporting); supervision (supporting); writing – review and editing (supporting). **Sean Yoder:** Conceptualization (supporting); formal analysis (supporting); methodology (supporting); project administration (supporting); resources (supporting); supervision (supporting); writing – review and editing (supporting). **Viswam S. Nair:** Conceptualization (equal); data curation (equal); formal analysis (equal); funding acquisition (equal); investigation (equal); methodology (equal); project administration (equal); resources (equal); supervision (equal); validation (equal); visualization (equal); writing – original draft (equal); writing – review and editing (equal).

## FUNDING INFORMATION

This research was supported by the Genomics & Bioinformatics Shared Resource RRID:SCR_022606 and the Specimen Processing Shared Resource, RRID:SCR_022619 of the Fred Hutch/University of Washington/Seattle Children's Cancer Consortium (P30 CA015704); the Tissue Core Facility at the H. Lee Moffitt Cancer Center & Research Institute, (P30‐CA076292); and the Cancer Center Support Grant of the Fred Hutchinson Cancer Center (VSN), NCI U01 CA253166 (VSN) and Roche (VSN).

## CONFLICT OF INTEREST STATEMENT

CS, PY, CZ, MPF, MF, and SY have nothing to declare. ET served as the advisory board member, consultant, speaker, and surgical investigator on several clinical trials that have been sponsored by Genentech‐Roche. AT served as the speaker for Pinnacle Biologics, Veracyte, and Biodesix. MS served as the consultant for Bristol Myers Squibb. Associate Editor for *Cancer Medicine*. VSN received research funding from Roche and served as an advisor in 2020.

## ETHICS APPROVAL STATEMENT

This study was approved by the Advarra Inc. Institutional Review Board according to the Declaration of Helsinki and the US Federal Policy for the Protection of Human Subjects.

## Supporting information


Figure S1.
Figure S2.

Table S1.
Table S2.
Table S3.
Table S4.
Table S5.
Table S6.
Table S7.
Table S8.
Table S9.
Table S10.
Table S11.

Data S1.

## Data Availability

The data that support the findings of this study are available in the supplementary material of this article. Additional data that support the findings of this study are available from the corresponding author upon reasonable request.
