# Supplementary figures and images for: Protocol optimization of a targeted sequencing panel for genomic profiling of bronchoalveolar lavage fluid in lung cancer
Source: Cancer Med. 2023 Aug 17;12(17):17632–7. doi: 10.1002/cam4.6380 (PMC10524020; doi:10.1002/cam4.6380)

S. Figure 1

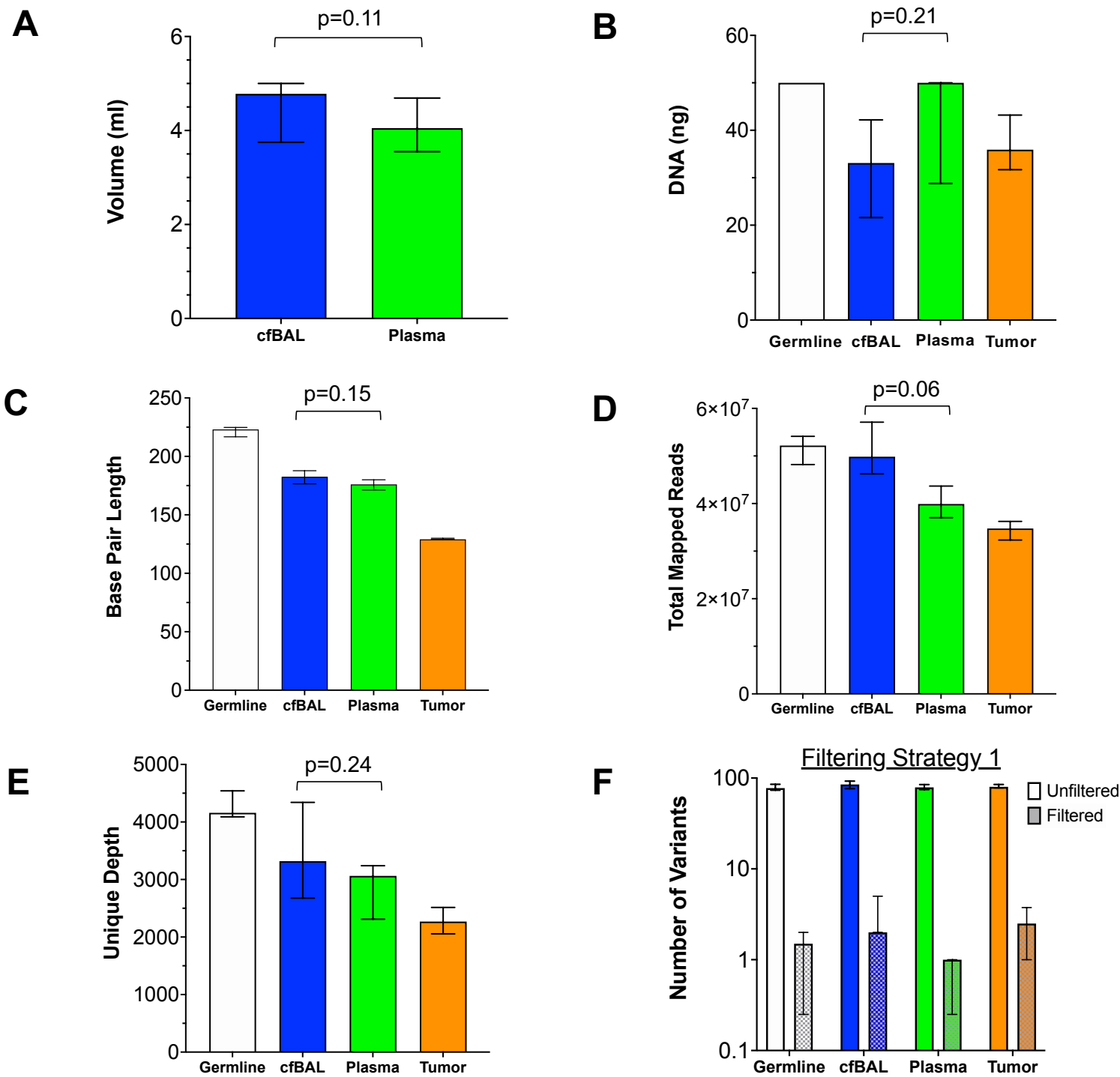

S. Figure 2

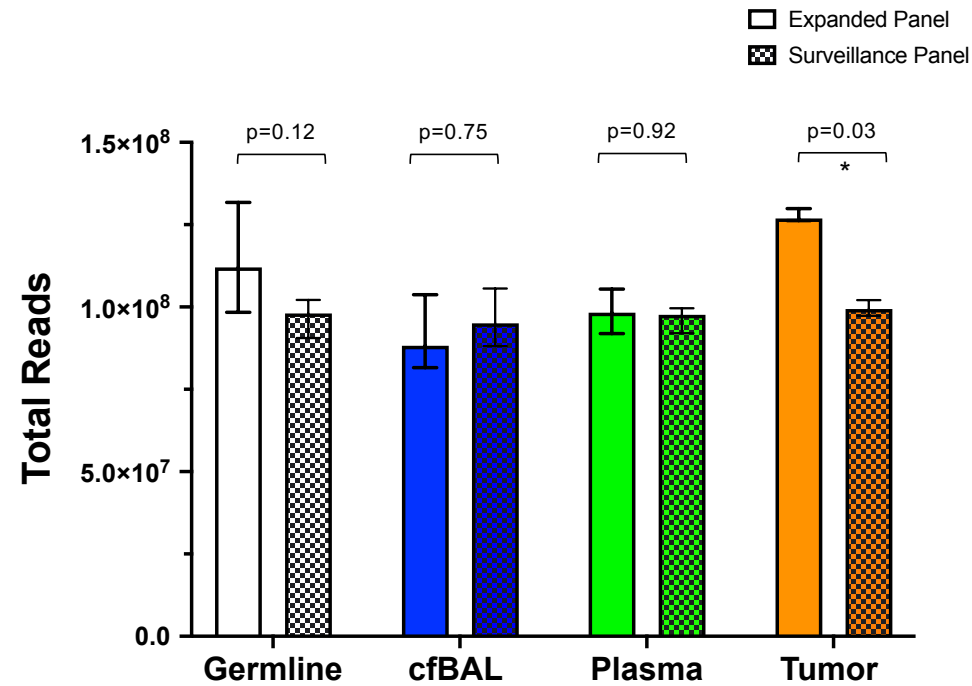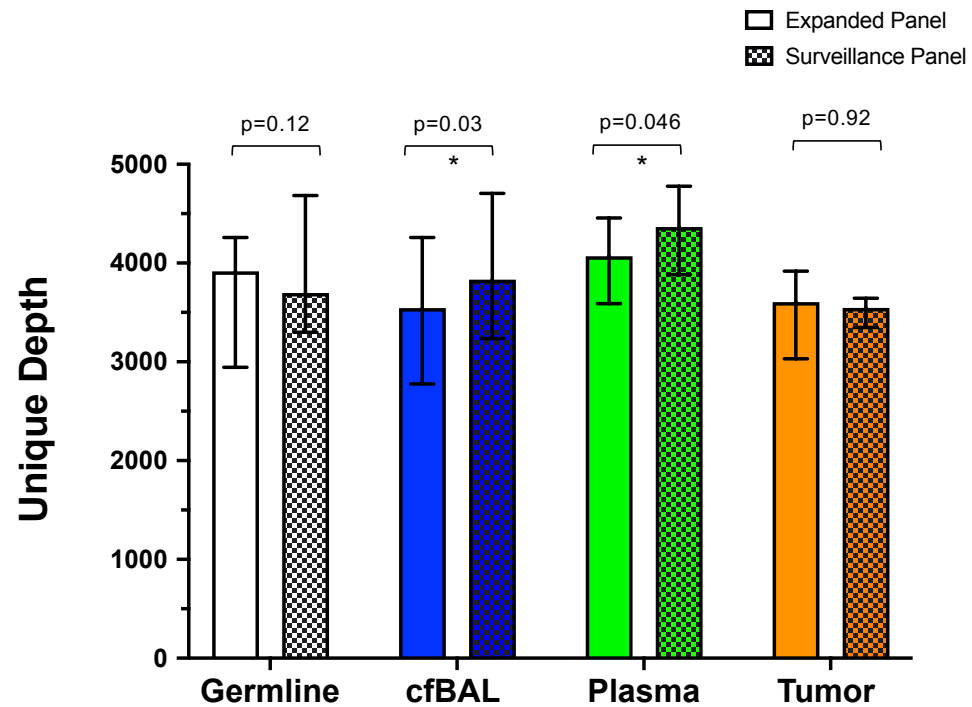

Supplement: Supplementary file 1 — Figure S1. Figure S2. [file CAM4-12-17632-s003.pdf]
