# Supplementary material for: Protocol optimization of a targeted sequencing panel for genomic profiling of bronchoalveolar lavage fluid in lung cancer
Source: Cancer Med. 2023 Aug 17;12(17):17632–7. doi: 10.1002/cam4.6380 (PMC10524020; doi:10.1002/cam4.6380)
Supplement: Supplementary file 3 — Data S1. [file CAM4-12-17632-s001.docx]

**Supplement to: Protocol optimization of a targeted sequencing panel for genomic profiling of bronchoalveolar lavage fluid in lung cancer**

Cassandra L. Sather,^1^ Pamela Yang,^1^ Chaomei Zhang,^2^ Matthew P. Fitzgibbon,^1^ Michelle Fournier, ^2^ Eric Toloza,^3^ Amit Tandon,^3†^ Matthew Schabath,^3,4^ Sean Yoder,^2^ Viswam S. Nair^5,6^

1. Fred Hutchinson Cancer Center, Shared Resources

2. H. Lee Moffitt Cancer Center & Research Institute, Tissue and Molecular Genomics Cores

3. H. Lee Moffitt Cancer Center & Research Institute, Department of Thoracic Oncology

4. H. Lee Moffitt Cancer Center & Research Institute, Department of Cancer Epidemiology

5. University of Washington, Division of Pulmonary, Critical Care & Sleep Medicine

6. Fred Hutchinson Cancer Center, Clinical Research Division

†Current Affiliation: Advent Health, Wesley Chapel, FL

**Supplemental Methods**

Enrollment and sample processing

Prior to biopsy or surgical resection, BAL was performed in the subsegment of interest by instilling and aspirating 120 ml of room temperature (RT) normal saline in two divided doses. Blood was procured prior to surgical resection into 10 ml K_2_EDTA tubes (Becton Dickinson) or 10 ml Streck™ tubes. BAL and blood were taken to the lab at RT. BAL was processed within 4 hours by serial centrifugation at 1200-1500g *1 0’ at 4^0^C to separate the cell pellet. The supernatant was aliquoted out into 5 ml cryovials and frozen at -80^0^C until DNA extraction. K_2_EDTA blood tubes were processed within 4 hours by centrifugation at 1200-1500 g * 10’ at 4^0^C to procure buffy coat and cell-free plasma. Streck™ blood tubes were processed within 4 hours by centrifugation at 300-500 g * 15’ at RT, collection of cells and then repeat centrifugation at 1200-1500 g * 10’ at 4^0^C to collect cell-free plasma that was aliquoted and stored at -80^0^C until DNA extraction. Plasma was extracted to cell-free DNA using the QIAmp Circulating Nucleic Acid Kit for fluids (Qiagen). Leukocytes from the buffy coat were extracted and archival tumor specimens were extracted to DNA using the DNAeasy Blood & Tissue Kit for cells (Qiagen). BAL cfDNA was fragmented (Covaris S2) without gDNA clean-up for 4 samples and with DNA cleanup for the remaining 6 samples. All research was IRB approved at the center of enrollment prior to initiation.

Description of AVENIO® panels used in this study

These are targeted, hybrid-capture based panels using probes spanning known cancer gene mutations. The expanded panel targets 77 genes with one or more probes spanning genes within each gene resulting in a targeted sequencing coverage area of 192 kb. The surveillance panel targets 197 genes with one or more probes spanning genes within each gene resulting in a sequencing coverage area of 198 kb (**S Table 1**). Based on information reported by the company and available on their website, these assays have been tested using cell free input material ranging from 5 to 100 ng. Sensitivity to detect variants increases with higher % Variant Allele Frequency (VAF) and increasing sequencing depth. For example, selecting 10 to 50 ng input material at 50 million paired-end reads that mirrors the current study, reported sensitivity in plasma for the expanded panel ranges from 56-65% at a threshold of 0.1% VAF to 100% at a threshold of 0.5% VAF.

The OAS provides two default variant output files. The unfiltered list includes a list of all variants detected in the specimen sample excluding only known publicly available germline variants not present in the loci of interest. The filtered list keeps somatic variants identified in COSMIC, TCGA or the loci of interest list and removes variants from dbSNP Common or those from the ExAC or 1000 genomes databases with > 0.1% VAF.

**Supplemental Figure 1. Sequencing metrics by biospecimen type**

(A) Input volume for cell-free BAL (cfBAL) and plasma carried forward to DNA extraction. (B) Median input DNA in nanograms for sequencing by biospecimen type. (C) Base-pair length during low coverage sequencing by biospecimen type. (D) Total mapped reads after low coverage sequencing by biospecimen type. Unique read depth after low coverage sequencing by biospecimen type. (F) Patient level unfiltered and filtered variants by biospecimen type in log_10_ scale after low coverage sequencing using Filtering Strategy 1 (FS 1). For all panels, median is displayed, and error bars depict the interquartile range. P-values are shown on top of each plot.

**Supplemental Figure 2. Sequencing metrics by targeted panel and biospecimen type**

Total mapped reads (top) and unique read depth (bottom) are illustrated binned by biospecimen and panel type for high coverage sequencing of six patients that underwent profiling using both the expanded and surveillance panels. cfBAL = cell-free BAL. Median is displayed, and error bars show the interquartile range. P-values are shown on top of each plot. An asterisk denotes a significant difference between groups.

**Supplemental Tables**

| Supplementary Table 1: | AVENIO® panel gene lists | | |  |
| --- | --- | --- | --- | --- |
| Supplementary Table 2: | Cohort characteristics |  |  |  |
| Supplementary Table 3: | Tumor quality scores assessed by PCR | |  |  |
| Supplementary Table 4: | Low coverage 77 gene panel summary statistics | | |  |
| Supplementary Table 5: | Low coverage 77 gene panel calls by fluid and cell type | | | |
| Supplementary Table 6: | High coverage 77 gene panel summary statistics | | |  |
| Supplementary Table 7: | High vs. low coverage 77 gene panel summary statistics | | |  |
| Supplementary Table 8: | High coverage 77 gene panel variant calls by fluid and cell type | | |  |
| Supplementary Table 9: | Cell-free BAL tumor-derived variants identified based on sequencing coverage and filtering strategy | | | |
| Supplementary Table 10: | High coverage 197 gene panel summary statistics | | | |
| Supplementary Table 11: | High coverage 197 gene panel variant calls by fluid and cell type | | | |
